# Orientational Structure and Electro-Optical Properties of Chiral Nematic Droplets with Conical Anchoring

**DOI:** 10.3390/molecules30244761

**Published:** 2025-12-12

**Authors:** Kristina A. Feizer, Mikhail N. Krakhalev, Vladimir Yu. Rudyak, Victor Ya. Zyryanov

**Affiliations:** 1 Kirensky Institute of Physics, Federal Research Center KSC SB RAS, Krasnoyarsk 660036, Russia; fka@iph.krasn.ru (K.A.F.); zyr@iph.krasn.ru (V.Y.Z.); 2 Institute of Engineering Physics and Radio Electronics, Siberian Federal University, Krasnoyarsk 660041, Russia; 3 Department of Condensed Matter, School of Physics and Astronomy, Tel Aviv University, Tel Aviv 69978, Israel; vurdizm@gmail.com

**Keywords:** chiral nematic liquid crystals, polymer dispersed liquid crystal, conical boundary conditions, twisted axial-bipolar structure, electro-optical properties, light transmittance

## Abstract

The polymer dispersed liquid crystals (PDLCs) with conical boundary conditions are considered. PDLC films with different values of the relative chirality parameter $N_{0}$ of chiral nematic droplets ranging from 0 to 1.32 are studied experimentally and theoretically. In flattened spheroid-shaped chiral nematic droplets, a twisted axial-bipolar structure is formed whose twist angle increases with rising $N_{0}$ value. Two stable states of the structure are revealed: one with the bipolar axis oriented perpendicular to the short axis of the spheroid and another with the bipolar axis oriented parallel to it. Applying a small voltage causes the bipolar axes of the chiral nematic droplets to reorient parallel to the electric field. The structure is unwound in strong electric fields, and the droplet order parameter reaches a high value of nearly 0.95. These features of the voltage-induced reorientation of the axial-bipolar structure explain the experimentally observed characteristic electro-optical properties of PDLC cells: high transmittance $T^{\max}≅0.90$ in the on-state and low control voltages of less than 35 V. The minimum transmittance of the PDLC cells decreases as the value of $N_{0}$ increases; for samples with $N_{0}\geq 0.60$, the contrast ratio exceeds 145.

## 1. Introduction

**Figure 1:**
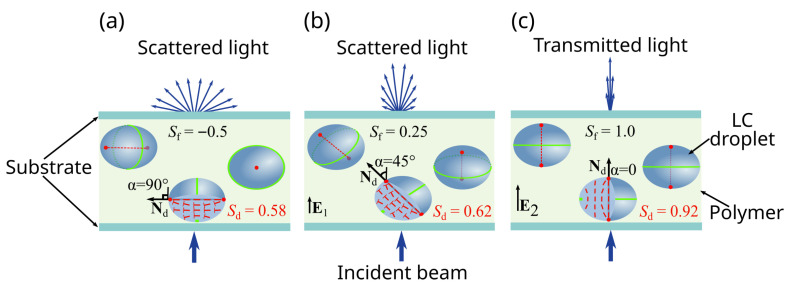
Sketch of the PDLC film switching from an initial opaque state (**a**) to a weakly scattering state (**b**) and a transparent state (**c**) under the action of low $E_{1}$ (**b**) and strong $E_{2}$ (**c**) electric fields, respectively. α is the angle between the incident beam and the $N_{d}$ direction of the symmetry axis of the LC droplets orientation structure. Blue arrows show incident and diffuse light.

Polymer dispersed liquid crystal (PDLC) is a composite material consisting of liquid crystal (LC) droplets randomly distributed within a polymer film [1]. The optical characteristics of the PDLC film depend on the ratio of the refractive indices of the polymer and liquid crystal, as well as the orientational structure formed in the LC droplets. The orientational structure is determined by the LC cavity size and shape, the boundary conditions, the LC material parameters, and the applied electric (or magnetic) field. An electric field applied to the PDLC film results in a variation of the LC orientation structure (Frederiks transitions), which leads to a change in the optical parameters of the entire film. This allows, for example, switching of the PDLC film from an initially light-scattering to a transparent state (Figure 1) [2,3,4], or vice versa [5,6]. This makes PDLC films ideal candidates for use in smart glasses [7,8], projection displays [9], dynamic three-dimensional (3D) holographic display [10], light-shaping films [11,12], micro-lenses [13], lasers [14], etc.

Recently, PDLC films have been investigated to improve their electro-optical characteristics, in particular, to reduce control voltages and response time, to increase contrast ratio (CR) and maximum transmittance value $T_{\max}$, and to improve their manufacturability. The enhancement of the electro-optical characteristics of PDLC can be accomplished through the selection of the most suitable film morphology or the optimal optical and dielectric parameters of the LC and polymer. For this purpose, the composition and fabrication conditions of the PDLC films are varied [15,16,17], the polymer matrix is modified with additives [18,19], and the PDLC is doped with nanoparticles [20,21,22], quantum dots [23] or dichroic dye [24,25].

The orientational structure formed in the LC droplets has a significant effect on the electro-optical parameters of the PDLC films. As a consequence, setting certain boundary conditions allows improving the electro-optical response of PDLC films. Numerous experimental and theoretical studies have been conducted on PDLC films with homeotropic [26,27] or tangential [28,29,30,31] boundary conditions in which the director is oriented perpendicular (θ=0) or parallel ($\theta =90^{\circ }$) to the droplet interface, respectively. Here, θ is the angle between the director and normal to the droplet surface at the polymer-LC interface. The radial structure transforms into an axial one when an electric field is applied to nematic droplets under homeotropic anchoring. This transformation increases the droplet order parameter $S_{d}=\langle \frac{1}{2}\left(3{\left(N_{d}n\right)}^{2}-1\right)\rangle$, which characterizes the orientation of the local LC director **n** relative to the symmetry axis of the droplet $N_{d}$. This results in the refractive indices of the LC droplets and the polymer matrix matching, causing the PDLC film to switch from an opaque to a transparent state. The PDLC films with homeotropic boundary conditions are characterized by low control voltages and low contrast ratio (*CR*). The bipolar orientational structure is formed in nematic droplets under tangential anchoring. The symmetry axis of the nematic droplets is reoriented parallel to the applied electric field. This increases the sampling order parameter $S_{f}=\langle \frac{1}{2}\left(3\cos^{2}\alpha -1\right)\rangle$, which characterizes the orientation of the droplets’ bipolar axes relative to the direction of the incident light, and α is the angle between the droplet bipolar axis $N_{d}$ and the incident light direction (Figure 1). This results in switching the PDLC film from a light-scattering to transparent state. The PDLC films with tangential boundary conditions are characterized by high contrast ratio and high control voltages.

The axial-bipolar configuration is formed in nematic droplets under conical anchoring ($\theta =40^{\circ }$). The application of a voltage leads to the orientation of the structure bipolar axis parallel to the electric field, resulting in the PDLC film switching from a light scattering to a transparent state [32]. Such PDLCs are characterized by low control voltage, high contrast ratio and high transmittance in the voltage-on state. However, after the switching off voltage, the PDLC film remains in the transparent state for a long time, which makes it difficult to use practically.

Chiral nematic liquid crystals (CLCs) are characterized by a helical director configuration in the free state. A twisted orientational structure is formed in CLC droplets, the type of which depends on the value of the relative chirality parameter $N_{0}=2d/p_{0}$, where *d* is the droplet diameter, and $p_{0}$ is the intrinsic helix pitch (the distance over which the director rotates by 2π in the free state) [33]. A twisted axial-bipolar structure is formed in CLC droplets under conical anchoring at $N_{0}<2.9$ [34]. Twisting of the orientational structure affects the droplet order parameter $S_{d}$, and consequently, the light scattering properties of the PDLC film. As a result, the switching-off time of the PDLC film containing the twisted axial-bipolar droplets with $N_{0}=0.32$ decreased to a few tens of milliseconds [35]. To date, the dependence of the electro-optical properties of PDLC films with conical anchoring on the $N_{0}$ value has not been performed.

In this work, the electro-optical properties of PDLC films based on CLC droplets under conical anchoring are studied experimentally and by numerical modeling. We examine PDLC films with five $N_{0}$ values ranging from 0 to 1.32. The electro-optical response of PDLC films is considered for the first and subsequent voltage turn-on and turn-off. The dependencies of the CLC orientational structures, the control voltages, CR, transmittance T(U), and the response times on the $N_{0}$ value are obtained and analyzed (see Table 1).

## 2. Results

### 2.1. CLC Droplets Structures

**Figure 2:**
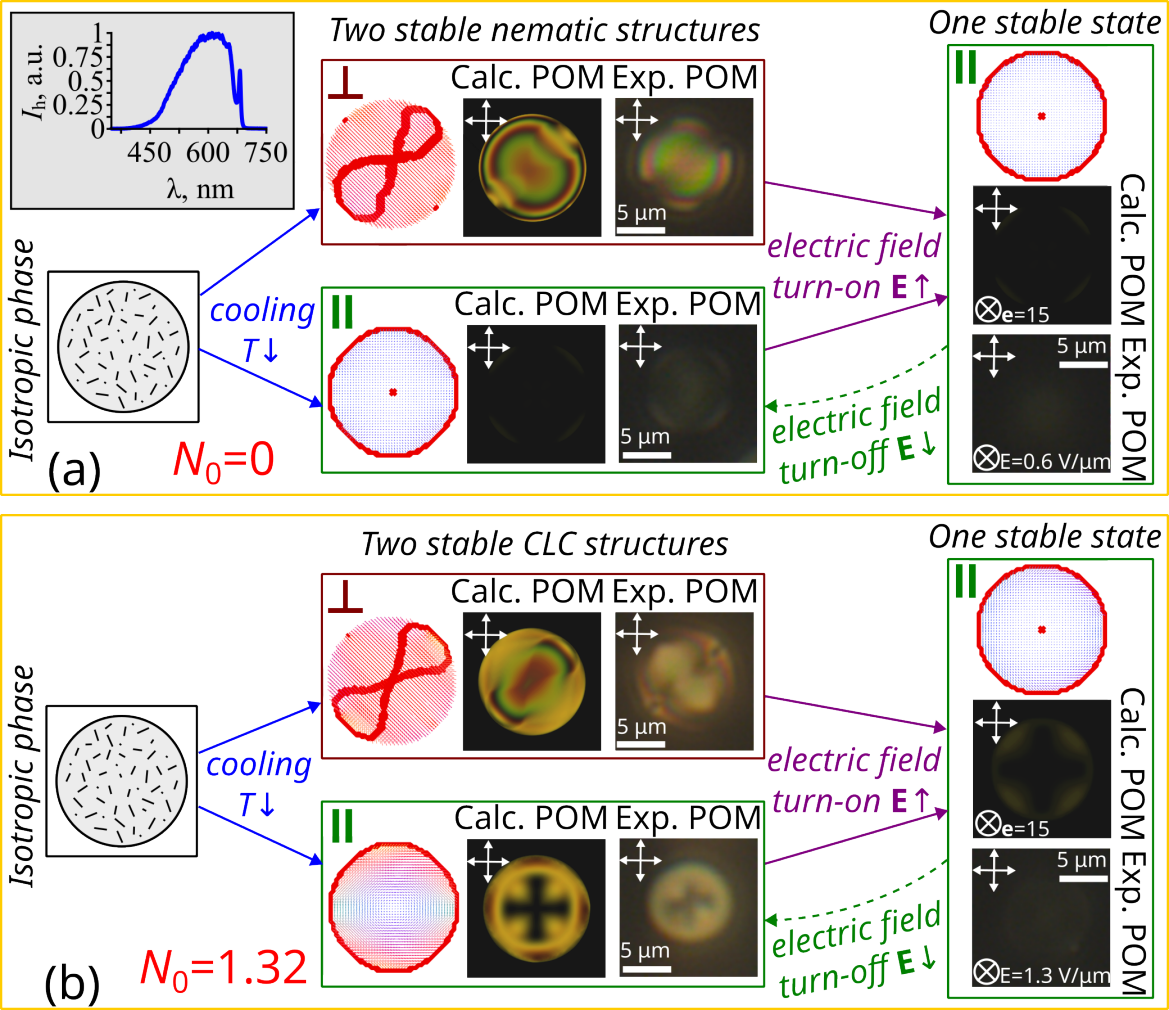
Calculated orientation structures and corresponding polarized optical microscopy (POM) images (Calc. POM) of droplets with $N_{0}=0$ (**a**) and $N_{0}=1.32$ (**b**), experimental POM photos (Exp. POM) of nematic ($N_{0}=0$) and CLC ($N_{0}=1.32$) droplets. The orientation of the bipolar axis of the CLC droplets is the same in the calculations and experiments. Before applying an electric field, the bipolar axis is initially oriented perpendicular (⊥) and parallel (‖) to the short axis of the droplet (middle column). LC droplets under the action of an electric field applied along the short axis of the droplet (last column). The short axis of the spheroid is perpendicular to the figure plane. The directions of the polarizers are shown by the double arrows in the calculated and experimental POM images. The insert in Figure 2a shows the emission spectrum $I_{h}$ of the halogen microscope lamp.

A twisted axial-bipolar structure characterized by two surface point defects located at the opposite poles of the droplet and a surface linear defect is formed in the CLC droplets in the form of a flattened spheroid at all studied values $N_{0}$ (Figure 2). The surface linear defect has a shape close to a ring and is located symmetrically relative to the point defects. Therefore, the structure can be considered axisymmetric, with the symmetry axis coinciding with the bipolar axis that connects the point defects. The calculation shows two stable states of the twisted axial-bipolar structure in an initial state, one with the bipolar axis oriented perpendicular to the short axis of the spheroid (marked with the index ⊥ in Figure 2a,b) and one with the bipolar axis oriented parallel to it (marked with the index ‖ in Figure 2a,b). Applying an electric field along the short spheroid axis leads to the reorientation of the bipolar axis parallel to it, and the unwinding of the structure up to a nearly homogeneous state in the strong fields. After switching off the electric field, only one stable state with a bipolar axis parallel to the short axis of the spheroid is realized. These changes in the orientational structure lead to variations in the transmittance T(U) of the PDLC film.

### 2.2. The Electro-Optical Response of PDLC Cells

**Figure 3:**
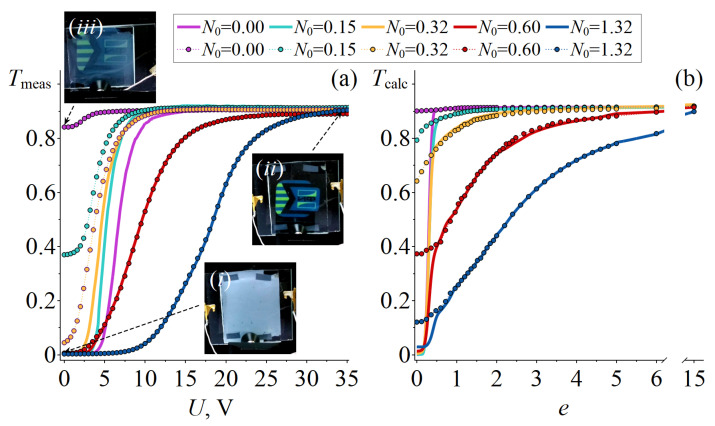
Dependencies of the transmittance $T_{\mathrm{meas}}$ on the applied voltage *U* measured at the first (solid lines) and subsequent (dotted lines with circles) voltage applications (**a**). The insets show macroscopic photos of the PDLC cell with $N_{0}=1.32$ at turn-off voltage (i) and at an applied voltage of 35 V (ii), as well as the PDLC cell with $N_{0}=0$ after turning-off the voltage (iii). Dependencies of the transmittance $T_{\mathrm{calc}}$ on the applied dimensionless field *e* calculated for the PDLC films with the droplets structure symmetry axes initially oriented parallel (solid lines) and perpendicular (dotted lines with circles) to the PDLC film (**b**).

In the experiment, the electro-optical properties of PDLC films during the first application of voltage differ from those at subsequent switching on–off of an electric field. Initially, all samples strongly scatter light, and the transmittance of the PDLC cells is $T_{\mathrm{meas}}^{0}≅0.003$ (Figure 3a, Table 2). When an electric field is first applied to samples with $N_{0}\leq 0.32$, the transmittance of PDLC cells rises sharply from $T_{\mathrm{meas}}^{0}≅0.003$ to $T_{\mathrm{meas}}^{\max}≅0.910$ as the applied voltage increases from 0 to U≅8.5 V (Figure 3a). For samples with $N_{0}\geq 0.6$, the slopes of the transmittance $T_{\mathrm{meas}}$ versus the applied voltage *U* dependencies are shallower. The maximum value of $T_{\mathrm{meas}}^{\max}≅0.90$ is achieved at voltages of U=27 V and U=35 V for PDLC cells with $N_{0}=0.60$ and $N_{0}=1.32$, respectively. When the voltage is turned off, the PDLC cells do not return to their initial scattering state (Figure 3a). The minimum value of transmittance $T_{\mathrm{meas}}^{\min}$ after switching off the voltage depends on the $N_{0}$ value and decreases from 0.84 to 0.003 as $N_{0}$ increases from 0 to 1.32 (Table 2). At subsequent voltage applications, the maximum transmittance value of CLC cells is achieved at the same voltage as when the electric field is first switched on (Figure 3a). Samples with $N_{0}\leq 0.32$ have high $T_{\mathrm{meas}}^{\min}$ values, which lead to low working contrast ratios $CR=\frac{T_{\mathrm{meas}}^{\max}}{T_{\mathrm{meas}}^{\min}}$ (Table 2). CR values are 148 and 303 for samples with $N_{0}=0.6$ and $N_{0}=1.32$, respectively. The control voltages of the studied PDLC cells are close to those of low-voltage PDLC cells based on fluorinated polymers [36] or polyacrylates with different alkyl chain structures [37,38]. Meanwhile, PDLC cells with conical anchoring exhibit higher $T^{\max}$ and CR values. High values of $T^{\max}≅90\%$ and CR>100 were achieved for the PDLC films doped with functionalized mesoporous silica materials, but higher voltages of U>50 V were used for this purpose [39]. High $T^{\max}≅90\%$ values and low control voltages of U≅15 V were achieved for PDLC cells with low contrast ratios of CR<40 [40,41].

The switching time of PDLC cells from opaque to transparent ($\tau_{\mathrm{on}}$) state, and vice versa ($\tau_{\mathrm{off}}$) decreases with increasing $N_{0}$. The switching times of PDLC cells with N≥0.32 were measured for subsequent voltage turn-ons and turn-offs. As the $N_{0}$ value increases from 0.32 to 1.32, the turn-on time $\tau_{\mathrm{on}}$ decreases slightly from 70±6 ms to 60±5 ms, while the turn-off time $\tau_{\mathrm{off}}$ is significantly reduced from 80±2 ms to 12±1 ms (Table 2).

Figure 3b shows the calculated dependencies of transmittance $T_{\mathrm{calc}}$ on the applied dimensionless field *e*. The short axes of the LC droplets are mainly perpendicular to the PDLC film plane, and therefore the direction of the incident light beam is mainly parallel to the short axis of the spheroids [42]. The $T_{\mathrm{calc}}\left(e\right)$ values were calculated for this case of the orientation of the incident beam relative to the short axis of the CLC droplets.

If the initial bipolar axes of the droplets are oriented perpendicular to the short axis of the spheroid, then the dependence of $T_{\mathrm{calc}}$ on the applied field *e* is threshold-type. The threshold field value increases with $N_{0}$. For droplets with $N_{0}\leq 0.32$, the transmittance of PDLC cells rises sharply from $T_{\mathrm{calc}}^{\min}=0.0005$ to $T_{\mathrm{calc}}^{\max}≅0.91$ as the field *e* increases from 0 to 2. Further increases in field *e* practically do not change the value of $T_{\mathrm{calc}}$. For the PDLC cell with $N_{0}\geq 0.6$, the transmittance of the PDLC film rises sharply from $T_{\mathrm{calc}}^{\min}=0.014$ to $T_{\mathrm{calc}}=0.41$ (at $N_{0}=0.60$), and from $T_{\mathrm{calc}}^{\min}=0.020$ to $T_{\mathrm{calc}}=0.25$ (at $N_{0}=1.32$). A further increase in the applied field *e* leads to a gradual rise in $T_{\mathrm{calc}}$ due to changes in the $S_{d}$ value. In high fields, the transmittance of PDLC cells reaches a value of $T_{\mathrm{calc}}^{\max}≅0.91$.

When the bipolar axis of the CLC droplets is initially oriented along the short axis of the spheroid, the dependence of transmittance $T_{\mathrm{calc}}$ on the applied field *e* is nonthreshold (Figure 3b). The minimum transmittance $T_{\mathrm{calc}}^{\min}$ decreases from 0.90 to 0.12 as the $N_{0}$ value increases from 0 to 1.32. In high fields, the transmittance of PDLC cells reaches a value of $T_{\mathrm{calc}}^{\max}≅0.91$ for all studied values of $N_{0}$.

The calculated dependencies $T_{\mathrm{calc}}\left(e\right)$ show that the electro-optical properties of PDLC films are determined by the initial orientation of the symmetry axes of the CLC droplets with a twisted axial-bipolar structure. This explains the difference observed in the electro-optical responses of PDLC cells measured at the first and subsequent voltage on–off cycles (Figure 3a). Let us consider in detail the electrically induced changes in the orientation of the droplets’ symmetry axes, as characterized by the sampling order parameter $S_{f}$, as well as the changes in orientation structure, as characterized by the droplet order parameter $S_{d}$.

## 3. Discussion

### 3.1. The Calculated Dependencies $T_{\mathrm{calc}}\left(e\right)$

**Figure 4:**
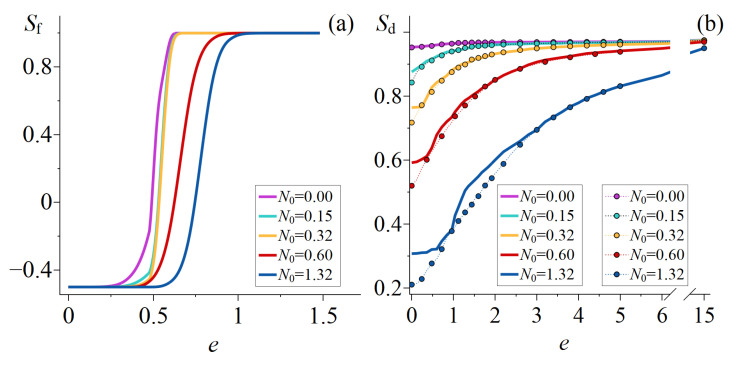
Calculated dependencies of the sampling order parameter $S_{f}$ (**a**) and the droplets order parameter $S_{d}$ (**b**) on the applied dimensionless field *e*. The droplets structure symmetry axis initially oriented parallel (solid lines) and perpendicular (dotted lines with circles) to the PDLC film.

If the initial bipolar axes of all droplets are oriented perpendicular to the short axis of the spheroid, then the sampling order parameter is equal to $S_{f}=-1/2$. The orientation of the bipolar axis remains practically unchanged at small values of the field *e* (Figure 4a). The threshold value of the field $e_{\mathrm{th}}$ corresponding to the beginning of the change in $S_{f}$ rises with the increasing value of $N_{0}$. For example, the threshold field for CLC droplets with $N_{0}=0$ is $e_{\mathrm{th}}≅0.32$, and it rises to $e_{\mathrm{th}}≅0.63$ for droplets with $N_{0}=1.32$. The bipolar axis reorientation process rapidly reaches saturation, at which $S_{f}=1$. The saturation field strength rises from $e_{S_{f}=1}=0.60$ to $e_{S_{f}=1}=1.00$ as $N_{0}$ increases from 0 to 1.32. As $N_{0}$ increases, the twist angle of the axial-bipolar structure rises, which consequently decreases the $S_{d}$ value (Figure 4b (solid lines)). In the off-state of an electric field, the value of $S_{d}$ decreases from 0.95 to 0.31 as $N_{0}$ increases from 0 to 1.32. The value of $S_{d}$ rises as the applied field *e* increases. The dependence of $S_{d}$ on the field *e* is nonthreshold. However, in the applied fields $e<e_{\mathrm{th}}$, the $S_{d}$ value changes slightly, and the main increase in $S_{d}$ occurs in the fields $e>e_{S_{f}=1}$. In strong fields, the value of $S_{d}$ is practically independent of $N_{0}$ and reaches a high value of $S_{d}≳0.95$ at e=15.

The threshold-type of the electro-optical response of PDLC cells results from the existence of the threshold field $e_{\mathrm{th}}$, and as the field value increases from $e_{\mathrm{th}}$ to $e_{S_{f}=1}$, the transmittance of the PDLC films rises sharply (Figure 3b, solid lines). For PDLC cells with N≤0.32, the primary change in transmittance $T_{\mathrm{calc}}\left(e\right)$ leads from the increase in $S_{f}$ (reorientation of the bipolar axis), while the change in $S_{d}$ slightly affects the $T_{\mathrm{calc}}\left(e\right)$ value. For PDLC cells with $N_{0}\geq 0.6$, the increase in $T_{\mathrm{calc}}$ induced by the electric field *e* is due to changes in both $S_{f}$ and $S_{d}$.

When the bipolar axis of the CLC droplets is initially oriented along the short axis of the spheroid (i.e., parallel to the direction of the incident beam), the sampling order parameter is $S_{f}=1$. In this case, the electro-optical response of the PDLC cells is due only to changes in $S_{d}$ (Figure 4b (dotted lines with circles)). The dependence of $S_{d}$ and, consequently, the transmittance $T_{\mathrm{calc}}$ on the applied field *e* is nonthreshold. The minimum $S_{d}$ value obtained at e=0 decreases from 0.95 to 0.21 as the $N_{0}$ increases from 0 to 1.32. Accordingly, the minimum transmittance $T_{\mathrm{calc}}^{\min}$ decreases from 0.90 to 0.12 as the $N_{0}$ value increases from 0 to 1.32. For all studied values of $N_{0}$, the droplet order parameter reaches $S_{d}≳0.95$ under strong field e=15, leading to an increase in transmittance up to $T_{\mathrm{calc}}^{\max}≅0.91$.

### 3.2. The Measured Dependencies $T_{\mathrm{meas}}\left(U\right)$

The observed electro-optical response of PDLC films can be explained by the features of the orientation of the bipolar axis of the CLC droplets. The shape of CLC droplets in PDLC films is known to be spheroids with a short axis oriented mainly perpendicular to the film plane [42]. Because the twisted axial-bipolar configuration has two stable states, a significant fraction of the droplets initially have their bipolar axis oriented parallel to the PDLC film. This leads to the opaque state of the PDLC cells in the initial state (Figure 3a). The high electric field applied to the sample orients the CLC droplets perpendicular to the film plane, resulting in a transparent state of PDLC cells with a high transmittance value $T_{\mathrm{meas}}^{\max}≅0.90$. Conical boundary conditions allow for the easy azimuthal “gliding” of the director at the interface under the influence of external factors [43,44]. As a result, the initial orientation of the bipolar axis of the CLC droplets is memory-free, and after the voltage is turned off, most of the CLC droplets remain oriented perpendicular to the PDLC film plane (see Figure 2). This leads to an increase in transmittance of the PDLC films in the off state (Figure 3a). The lower experimental transmittance $T_{\mathrm{meas}}^{\min}$ (Figure 3a) compared to the transmittance $T_{\mathrm{calc}}^{\min}$ (Figure 3b) calculated for the case of orientation of the droplets’ bipolar axis perpendicular to the PDLC film is due to the fact that in experimental samples the CLC droplets are not an ideal spheroid shape [1]. As a result, the bipolar axis of some droplets is not perpendicular to the PDLC film ($S_{f}<1$) when it is in an off state. This leads to a decrease in transmittance $T_{\mathrm{meas}}^{\min}$ that depends strongly on the $S_{d}$ value and decreases as the $N_{0}$ value increases.

The investigated PDLC cells are characterized by a high transmittance $T_{\mathrm{meas}}^{\max}≅0.90$ in the switched-on state. The PDLC cell can be adjusted to the required parameters by selecting an appropriate $N_{0}$ value without changing the composition and/or morphology of the film. For example, an increase in the $N_{0}$ value results in a higher contrast ratio and a lower slope of the T(U) curve. If control voltages need to be reduced, then PDLC films with smaller $N_{0}$ values should be used.

The studied PDLC cells are characterized by a 40-degree director tilt angle at the polymer-LC interface. A wide range of director tilt angles is available under conical boundary conditions. This provides an opportunity to improve the electro-optical properties of PDLC films by choosing the tilt angle, for example, by varying the polymer composition [45]. The variation in the $N_{0}$ value allows for control of the electro-optical parameters of the PDLC cell, including the minimum light transmittance, the control voltages, and the slope of the dependence T(U). As a result, it is possible to produce thermo- and/or photo-controlled PDLC films using thermosensitive or photosensitive cholesteric materials [46,47]. In this case, the value of $N_{0}$ and consequently the light transmittance *T* can be controlled by changes in temperature or exposure to light. It can lead to the expansion of practical application and functionality of PDLC films.

## 4. Materials and Methods

### 4.1. Samples Fabrication

Experimental studies were carried out for PDLC films based on polymer poly(isobutyl methacrylate) (PiBMA) (Sigma Aldrich, St. Louis, MO, USA) and nematic LN-396 (Belarusian State Technological University, Minsk, Belarus) doped with cholesteryl acetate (Sigma Aldrich). Five PDLC cells were investigated. The concentrations of cholesteryl acetate in LN-396 were 0%, 0.5%, 1.0%, 2.0%, and 4.0% (Table 3). The CLC:PiBMA weight ratio was 60:40. The thickness of the PDLC films was determined by 20 μm Teflon spacers. PDLC cells were manufactured using combined solvent-induced phase separation (SIPS) and thermal-induced phase separation (TIPS) technology [48]. The average diameter of the CLC droplets was 2.3 ± 0.1 μm. This corresponds to the average values of the relative chirality parameter of 0, 0.15±0.01, 0.32±0.02, 0.60±0.03, and 1.32±0.05 (Table 3). To study the orientation structures of the LC droplets and their response to an electric field, PDLC cells with an average droplet diameter of 10 μm were manufactured.

### 4.2. Electro-Optical Measurement

A standard technique for measuring the intensity of a forward-transmitted He-Ne (λ=632 nm) laser (Linos, Feldkirchen, Germany) beam was used to study the electro-optical response of PDLC cells [1]. The intensity of the laser beam passed through the PDLC cell was measured using a PDA100A-EC silicon detector (Thorlabs, Newton, NJ, USA). The detector was equipped with an iris stop that had an angular size of 50 min, which corresponded to the angular size of the unscattered laser beam. A digital multimeter 34465A (Keysight, Santa Rosa, CA, USA) was used to measure the detector’s signal. The transmittance was equal to $T_{\mathrm{meas}}=I/I_{0}$, where *I* is the intensity of light passed through the PDLC cell and $I_{0}$ is the intensity of the incident beam (measured without the PDLC cell). An AC voltage of 1 kHz frequency and variable amplitude was applied to the PDLC cell by the signal generator AFG-72225 (Instek, New Taipei City, Taiwan) combined with the amplitude amplifier AVA-1810 (Aktakom, Moscow, Russia). The $T_{\mathrm{meas}}\left(U\right)$ dependencies measured for several cycles of applying voltage to the PDLC cell differ by less than $0.001\cdot T_{\mathrm{meas}}\left(U\right)$. The CLC droplet structures were analyzed using the Axio Imager.M1m (Zeiss, Oberkochen, Germany) polarizing optical microscope (POM).

### 4.3. Calculating Droplets Structures

The droplet structures were calculated using Frank’s extended elastic continuum approach with annealing optimization using the Monte-Carlo method [49]. The shape of the CLC droplets was taken in the form of a flattened spheroid with an aspect ratio of x:y:z = 1:1:0.8 [42]. The ratio between the elasticity constants was $K_{11}$:$K_{22}$:$K_{33}$ = 1:0.48:1.63. The linear energy density of the disclination core was taken to be $f_{coreline}=2.75K_{11}$. The dimensionless polar anchoring energy was $\mu =\frac{WD}{2K_{11}}=7.92$, where $D={\left(d\cdot d\cdot 0.8d\right)}^{\frac{1}{3}}=2.16$ μm is the effective diameter of the droplet, $W=6.5\cdot 10^{-5}$ J m^−2^ is the surface anchoring energy, measured experimentally by the electric field method [50]. Conical boundary conditions with an angle of $40^{\circ }$ between the director and the droplet surface normal were used in the CLC droplet calculation [32]. The dimensionless field $e=\left|E_{\mathrm{appl}}\right|D{\left(\varepsilon_{0}\Delta \varepsilon /8K_{11}\right)}^{1/2}$ was used to model the effect of an electric field on the orientational structure of the CLC [49], where $E_{\mathrm{appl}}$ is the applied electric field strength, and Δε=10.2 is the dielectric permittivity anisotropy of the LC, $\varepsilon_{0}$ is the electric constant. The values of the relative chirality parameter of the CLC droplet were $N_{0}=0$, $N_{0}=0.15$, $N_{0}=0.32$, $N_{0}=0.6$, and $N_{0}=1.32$. The color POM textures of CLC droplets were generated by merging textures calculated using the Jones matrix technique for ten wavelengths ranging from 400 to 700 nm, with an interval of 33.33 nm between each wavelength. The calculations were made taking into account the emission spectrum of the halogen microscope lamp used (see insert in Figure 2a).

### 4.4. Calculating Light Transmittance

Since πd/λ>>1, the transmittance of the PDLC cell was calculated using the anomalous diffraction approach, taking into account the light reflection on the glass substrates as follows [51,52,53]:

(1) $$T_{\mathrm{calc}}={\left(1-0.04\right)}^{2}\exp\left(-N_{\nu }\sigma h\right),$$

where the number of droplets per unit volume $N_{\nu }=0.12$ μm^−3^ is calculated based on the parameters of the studied CLC cells, σ is the total cross-section of the droplet, h=20 μm is the thickness the studied PDLC film. The total droplet cross-section value is determined by the ordinary $n_{\mathrm{do}}$ and extraordinary $n_{\mathrm{de}}$ refractive indices of the CLC droplet and the sampling order parameter $S_{f}$. The refractive indices of the CLC droplet depend on the droplet order parameter $S_{d}$, as well as on the refractive indices of the polymer $n_{p}$ and the LC $n_{⊥}$, $n_{\|}$ (indices *⊥* and ‖ correspond to the polarization of light perpendicular and parallel to the director, respectively).

The values of Lagrange polynomials of the second kind $S_{f}$ and $S_{d}$ were determined based on the calculated structures of the CLC in the droplets. The parameter $S_{f}=\langle \frac{1}{2}\left(3\cos^{2}\alpha -1\right)\rangle$ characterizes the orientation of the bipolar axis of the droplets relative to the direction of incident light. Here, α is the angle between the droplet bipolar axis $N_{d}$ and the incident light direction. The transmittance $T_{\mathrm{calc}}$ was calculated by approximating that all CLC droplets in the film respond to the electric field in the same way. In other words, the angle α(e) is the same for all CLC droplets in the film. If the bipolar axes of the CLC droplets lie in the film plane, the sampling order parameter is $S_{f}=-0.5$ (Figure 1a). When a voltage is applied, the CLC droplets tend to align their bipolar axes with the electric field [32]. This increases the $S_{f}$ value, which is equal to 1 in a strong electric field (Figure 1b,c). The parameter $S_{d}=\langle \frac{1}{2}\left(3{\left(N_{d}n\right)}^{2}-1\right)\rangle$ characterizes the orientation of the local LC director **n** relative to the symmetry axis of the droplet $N_{d}$. When the LC director is randomly oriented within the droplet, the droplet order parameter is $S_{d}=0$. It is $S_{d}=1$ when the director is parallel to $N_{d}$ throughout the entire CLC droplet. The refractive indices of the nematic LN-396 ($n_{⊥}=1.520$ and $n_{\|}=1.720$) [54] and the polymer matrix ($n_{p}=1.518$) [32] were used to calculate the $S_{d}$ values.

## 5. Conclusions

PDLC cells with different values of the relative chirality parameter $N_{0}$ of chiral nematic droplets with conical boundary conditions have been studied. It has been shown that an axisymmetric twisted axial-bipolar structure is formed in the flattened spheroid-shaped CLC droplets. Two stable states of the twisted axial-bipolar structure have been revealed: one with the bipolar axis oriented perpendicular to the short axis of the spheroid and another with the bipolar axis oriented parallel to it. As the $N_{0}$ value rises, the twist angle of the structure increases, leading to a decrease in the order parameter $S_{d}$ of the droplets. Applying a small voltage causes the bipolar axes of the CLC droplets to reorient along the electric field. Increasing the voltage further results in the unwinding of the structure, and $S_{d}$ reaches a high value of nearly 0.95 in strong applied electric fields. This allows switching the PDLC cells from an opaque state to a transparent state with a high transmittance $T^{\max}≅0.90$. The minimum transmittance of the PDLC cells decreases as the value of $N_{0}$ increases. The studied PDLC cells are characterized by low control voltages of less than 35 V, and for samples with $N_{0}=0.60$ and $N_{0}=1.32$, the contrast ratio is more than 145. The obtained results can be used to expand the range of applications and improve the functionality of PDLC films.

## Figures and Tables

**Table 1:** Parameters of the PDLC cells and the CLC droplets, which are measured and calculated in the paper.

Parameter	Definition
$N_{0}=\frac{2d}{p_{0}}$	The relative chirality parameter
$T_{\mathrm{meas}}^{0}$	The measured transmittance of the PDLC cell in the initial state (before applying voltage)
$T_{\mathrm{meas}}^{\max}$	The measured transmittance of the PDLC cell at the turned-on high voltage
$T_{\mathrm{meas}}^{\min}$	The measured transmittance of the PDLC cell at the turned-off high voltage
$CR=\frac{T_{\mathrm{meas}}^{\max}}{T_{\mathrm{meas}}^{\min}}$	The contrast ratio
$\tau_{\mathrm{on}}$	The measured turn-on time is the time to switch to the transparent state
$\tau_{\mathrm{off}}$	The measured turn-off time is the time to switch to the opaque state
$T_{\mathrm{calc}}$	The calculated transmittance of the PDLC cell
$S_{f}=\langle \frac{1}{2}\left(3\cos^{2}\alpha -1\right)\rangle$	The calculated sampling order parameter
$S_{d}=\langle \frac{1}{2}\left(3{\left(N_{d}n\right)}^{2}-1\right)\rangle$	The calculated droplet order parameter

**Table 2:** The average value of the relative chirality parameter $N_{0}$; the measured transmittance $T_{\mathrm{meas}}^{0}$ of the PDLC cell in the initial state (before applying voltage); the measured minimum transmittance $T_{\mathrm{meas}}^{\min}$ at the turned-off voltage; the measured maximum transmittance $T_{\mathrm{meas}}^{\max}$ at the turned-on high voltage; the contrast ratio *CR*, the turn-on $\tau_{\mathrm{on}}$ and turn-off $\tau_{\mathrm{off}}$ times.

$N_{0}$	$T_{\mathrm{meas}}^{0}$	$T_{\mathrm{meas}}^{\min}$	$T_{\mathrm{meas}}^{\max}$	*CR*	$\tau_{\mathrm{on}}$, ms	$\tau_{\mathrm{off}}$, ms
0.0	0.0027	0.840	0.91	1.08	–	–
0.15	0.0032	0.370	0.91	2.46	–	–
0.32	0.0031	0.045	0.91	20	70	80
0.60	0.0032	0.006	0.89	148	68	35
1.32	0.0029	0.003	0.91	303	60	12

**Table 3:** The concentration of cholesteryl acetate *C*, the average diameter of the CLC droplets *d*, and the average value of the relative chirality parameter $N_{0}$ at the studied PDLC films.

*C*,%	*d*, μm	$N_{0}$ Value
0.0	2.3	0.0
0.5	2.2	0.15
1.0	2.3	0.32
2.0	2.2	0.60
4.0	2.4	1.32

## Data Availability

The data presented in this study are available on request from the corresponding author.

## References

[1] Drzaic P.S. (1995). Liquid Crystal Dispersions.

[2] Jinqian L., Zhao Y., Gao H., Wang D., Miao Z., Cao H., Yang Z., He W. (2021). Polymer dispersed liquid crystals doped with CeO_2_ nanoparticles for the smart window. Liq. Cryst..

[3] Meng X., Li J., Lin Y., Liu X., Zhao J., Li D., He Z. (2022). Periodic Electro-Optical Characteristics of PDLC Film Driven by a Low-Frequency Square Wave Voltage. Crystals.

[4] Mahar M., Muhammad J., Ali M., Mangi K.H., Murad A., Iqbal M. (2022). Preparation of silver nanoparticles (AgNPs)-doped epoxy-based thin PDLC films (smart glass). Polym. Bull..

[5] Sharma V., Kumar P., Jaggi C., Malik P., Raina K. (2019). Preparation and electrooptic study of reverse mode polymer dispersed liquid crystal: Performance augmentation with the doping of nanoparticles and dichroic dye. J. Appl. Polym. Sci..

[6] Yin S., Ge S., Zhao Y., Lu W., Ma H., Sun Y. (2023). Influence of different small styryl molecules on electro-optical characteristics of reverse-mode polymer stabilised cholesteric liquid crystal devices. Liq. Cryst..

[7] Islam M.S., Chan K.Y., Thien G.S.H., Low P.L., Lee C.L., Wong S.K., Noor E.E.M., Au B.W.C., Ng Z.N. (2023). Performances of Polymer-Dispersed Liquid Crystal Films for Smart Glass Applications. Polymers.

[8] Deng Y., Yang Y., Xiao Y., Zeng X., Xie H.L., Lan R., Lanying Z., Yang H. (2024). Annual Energy-Saving Smart Windows with Actively Controllable Passive Radiative Cooling and Multimode Heating Regulation. Adv. Mater..

[9] Agarwal S., Srivastava S., Joshi S., Tripathi S., Singh B.P., Pandey K.K., Manohar R. (2025). A Comprehensive Review on Polymer-Dispersed Liquid Crystals: Mechanisms, Materials, and Applications. ACS Mater. Au.

[10] Sun S., Li J., Li X., Zhao X., Li K., Chen L. (2024). Dynamic 3D metasurface holography via cascaded polymer dispersed liquid crystal. Microsyst. Nanoeng..

[11] Jiang J., McGraw G., Ma R., Brown J., Yang D.K. (2017). Selective scattering polymer dispersed liquid crystal film for light enhancement of organic light emitting diode. Opt. Express.

[12] He Z., Yin K., Hsiang E.L., Wu S.T. (2020). Volumetric light-shaping polymer-dispersed liquid crystal films for mini-LED backlights. Liq. Cryst..

[13] Zhou L., Liu S., Miao X., Xie P., Sun N., Xu Z., Zhong T., Zhang L., Shen Y. (2023). Advancements and Applications of Liquid Crystal/Polymer Composite Films. ACS Mater. Lett..

[14] Hsiao V.K., Lu C., He G.S., Pan M., Cartwright A.N., Prasad P.N., Jakubiak R., Vaia R.A., Bunning T.J. (2005). High contrast switching of distributed-feedback lasing in dye-doped H-PDLC transmission grating structures. Opt. Express.

[15] Ahmad F.K., Jamil M.M.A., Lee J.W., Kim S.R., Jeon Y.J. (2016). The effect of UV intensities and curing time on polymer dispersed liquid crystal (PDLC) display: A detailed analysis study. Electron. Mater. Lett..

[16] Kalkar A., Kunte V., Bhamare S. (2008). Electrooptic studies on polymer-dispersed liquid-crystal composite films. III. Poly(methyl methacrylate-co-butyl acrylate)/E7 and poly(methyl methacrylate-co-butyl acrylate)/E8 composites. J. Appl. Polym. Sci..

[17] Koo J.J., No Y.S., Jeon C., Kim J.H. (2008). Improvement of Electro-Optic Properties in PDLC Device by Using New Cross-Linker for the Control of the Contrast Ratio, Response Time and Driving Voltage. Mol. Cryst. Liq. Cryst..

[18] Qinqin W., Wang Y. (2017). Low driving voltage ITO doped polymer-dispersed liquid crystal film and reverse voltage pulse driving method. Appl. Opt..

[19] Cupelli D., Nicoletta F., Filpo G., Chidichimo G., Fazio A., Gabriele B., Salerno G. (2004). Fine adjustment of conductivity in polymer-dispersed liquid crystals. Appl. Phys. Lett..

[20] Ahmad F.K., Jeon A., Jeon Y.J., Jamil M.M.A. (2021). A novel technique of fabrication of nanoparticle acrylate doped polymer dispersed liquid crystal (PDLC) film. J. Dispers. Sci. Technol..

[21] Kumari A., Sinha A. (2020). Role of BaTiO_3_ nanoparticles on electro-optic performance of epoxy-resin-based PDLC devices. Liq. Cryst..

[22] Mhatre M., Katariya-Jain A., Deshmukh R. (2023). Improved electro-optical and dielectric properties of polymer dispersed liquid crystal doped with disperse dye red 1 and carbon nanoparticles. Liq. Cryst..

[23] Hsu C.C., Chen Y.X., Li H.W., Hsu J.S. (2016). Low switching voltage ZnO quantum dots doped polymer-dispersed liquid crystal film. Opt. Express.

[24] Deshmukh R., Katariya-Jain A. (2014). The complete morphological, electro-optical and dielectric study of dichroic dye-doped polymer-dispersed liquid crystal. Liq. Cryst..

[25] Zhao C., Hu Y., Xu J., Yu M., Zou C., Qian W., Gao Y., Yang H. (2023). Research on the Morphology, Electro-Optical Properties and Mechanical Properties of Electrochromic Polymer-Dispersed Liquid Crystalline Films Doped with Anthraquinone Dyes. Crystals.

[26] Boussoualem M., Ismaili M., Roussel F. (2014). Influence of surface anchoring conditions on the dielectric and electro-optical properties of nematic droplets dispersed in a polymer network. Soft Matter.

[27] Sharma V., Kumar P. (2021). Electric Field Dependent Textural Variation inside the Liquid Crystal Droplets with Homeotropic Alignment. J. Phys. Conf. Ser..

[28] Liang Z.Y., Tu C.Y., Yang T.H., Liu C.K., Cheng K.T. (2018). Low-Threshold-Voltage and Electrically Switchable Polarization-Selective Scattering Mode Liquid Crystal Light Shutters. Polymers.

[29] Malik P., Raina K. (2004). Droplet orientation and optical properties of polymer dispersed liquid crystal composite films. Opt. Mater..

[30] Deshmukh R.R., Katariya-Jain A. (2016). Novel techniques of PDLC film preparation furnishing manifold properties in a single device. Liq. Cryst..

[31] Kumar P., Sharma V., Jaggi C., Raina K.K. (2017). Dye-dependent studies on droplet pattern and electro-optic behaviour of polymer dispersed liquid crystal. Liq. Cryst..

[32] Krakhalev M.N., Prishchepa O.O., Sutormin V.S., Zyryanov V.Y. (2019). Polymer dispersed nematic liquid crystal films with conical boundary conditions for electrically controllable polarizers. Opt. Mater..

[33] Seč D., Porenta T., Ravnik M., Žumer S. (2012). Geometrical frustration of chiral ordering in cholesteric droplets. Soft Matter.

[34] Gardymova A., Krakhalev M., Zyryanov V. (2020). Optical Textures and Orientational Structures in Cholesteric Droplets with Conical Boundary Conditions. Molecules.

[35] Feizer K., Krakhalev M., Zyryanov V. (2022). Electrically induced optical and structural response of cholesteric and nematic droplets with conical boundary conditions. Liq. Cryst. Their Appl..

[36] Zhang Z., He X., Zhang L., Xu J., Yuan B., Chen C., Zou C., Wang Q., Gao Y., Yu M. (2024). A novel low-voltage fast-response electrically controlled dimming film based on fluorinated PDLC for smart window applications. Chem. Eng. J..

[37] Yu M., Xu J., Wang T., Zhang L., Wei H., Zou C., Gao Y., Yang H. (2022). Effects of acrylate monomers with different alkyl chain structure on the electro-optical properties and microstructure of polymer dispersed liquid crystals. J. Appl. Polym. Sci..

[38] Sun N., Zhang Z., Yang H. (2025). Effects of Diverse Acrylates on the Electro-Optical Performance of Polymer-Dispersed Liquid Crystal Films. Molecules.

[39] Zhao J., He Z., Yu P., Ma Z., Chen B., Yao W., Miao Z. (2025). Functionalized mesoporous silica composites for the modulation of polymer dispersed liquid crystals. Surfaces Interfaces.

[40] Ren Y., Hu W. (2025). Effects of Multi-Fluorinated Liquid Crystals with High Refractive Index on the Electro-Optical Properties of Polymer-Dispersed Liquid Crystals. Materials.

[41] Lin Y.H., Ren H., Wu S.T. (2004). High contrast polymer-dispersed liquid crystal in a 90° twisted cell. Appl. Phys. Lett..

[42] Wu B.G., Erdmann J.H., Doane J.W. (1989). Response times and voltages for PDLC light shutters. Liq. Cryst..

[43] Ramdane O.O., Auroy P., Forget S., Raspaud E., Martinot-Lagarde P., Dozov I. (2000). Memory-free conic anchoring of liquid crystals on a solid substrate. Phys. Rev. Lett..

[44] Kostikov D.A., Krakhalev M.N., Prishchepa O.O., Zyryanov V.Y. (2021). Nematic Structures under Conical Anchoring at Various Director Tilt Angles Specified by Polymethacrylate Compositions. Polymers.

[45] Yan B., He J., Du X., Zhang K., Wang S., Pan C., Wang Y. (2009). Control of liquid crystal droplet configuration in polymer dispersed liquid crystal with macro-iniferter polystyrene. Liq. Cryst..

[46] Balenko N., Shibaev V., Bobrovsky A. (2024). Polymer dispersed cholesteric liquid crystals with combined photo- and mechanochromic response. J. Mol. Liq..

[47] Yang Z.H., Hsiao Y.C., Shen D., Lee W. (2017). A thermally tunable narrowband selector based on a chiral nematic containing a binary thermosensitive chiral dopant mixture. Mol. Cryst. Liq. Cryst..

[48] Drzaic P.S., Scheffer T.J. (1995). Liquid Crystal Dispersions. J. Soc. Inf. Disp..

[49] Rudyak V.Y., Emelyanenko A.V., Loiko V.A. (2013). Structure transitions in oblate nematic droplets. Phys. Rev. E.

[50] Nastishin Y., Polak R., Shiyanovskii S., Lavrentovich O. (1999). Determination of nematic polar anchoring from retardation versus voltage measurements. Appl. Phys. Lett..

[51] Žumer S. (1988). Light scattering from nematic droplets: Anomalous-diffraction approach. Phys. Rev. A.

[52] Kelly J.R., Palffy-Muhoray P. (1994). The Optical Response of Polymer Dispersed Liquid Crystals. Mol. Cryst. Liq. Cryst..

[53] Simoni F., Francescangeli O. (2000). Optical Properties of Polymer-dispersed Liquid Crystals. Int. J. Polym. Mater. Polym. Biomater..

[54] Sutormin V.S., Krakhalev M.N., Timofeev I.V., Bikbaev R.G., Prishchepa O.O., Zyryanov V.Y. (2021). Cholesteric layers with tangential-conical surface anchoring for an electrically controlled polarization rotator. Opt. Mater. Express.

